# Supplementary material for: Oleracone F Alleviates Cognitive Impairment and Neuropathology in APPswe/PSEN1dE9 Mice by Reducing the Expression of Vascular Cell Adhesion Molecule and Leukocyte Adhesion to Brain Vascular Endothelial Cells
Source: Int J Mol Sci. 2023 Jan 20;24(3):2056. doi: 10.3390/ijms24032056 (PMC9916962; doi:10.3390/ijms24032056)
Supplement: Supplementary file 1 [file ijms-24-02056-s001.zip › supplementary_Oleracones_20221227.pptx]

## Slide 1
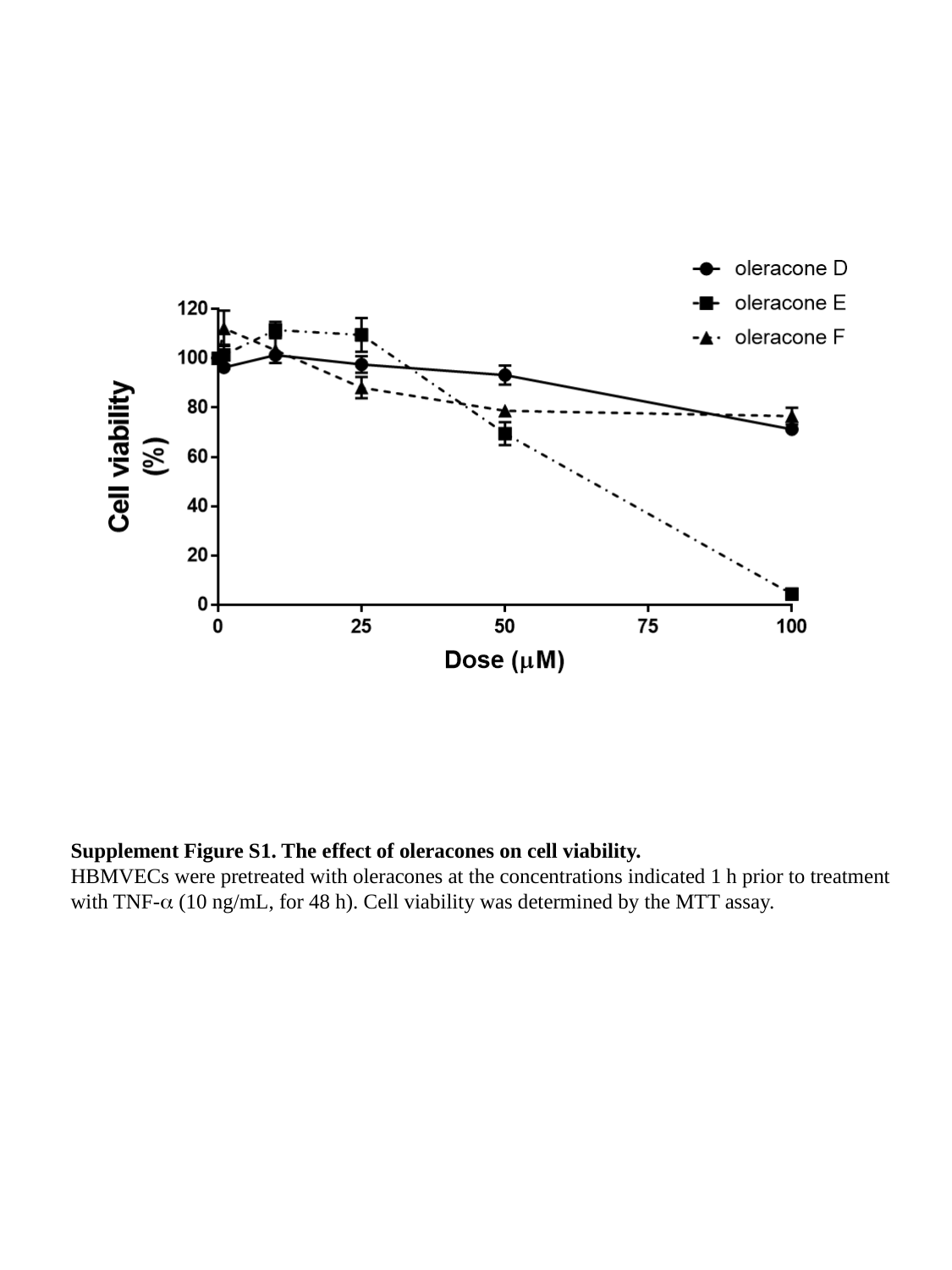

Supplement Figure S1. The effect of oleracones on cell viability.
HBMVECs were pretreated with oleracones at the concentrations indicated 1 h prior to treatment with TNF-a (10 ng/mL, for 48 h). Cell viability was determined by the MTT assay.

## Slide 2
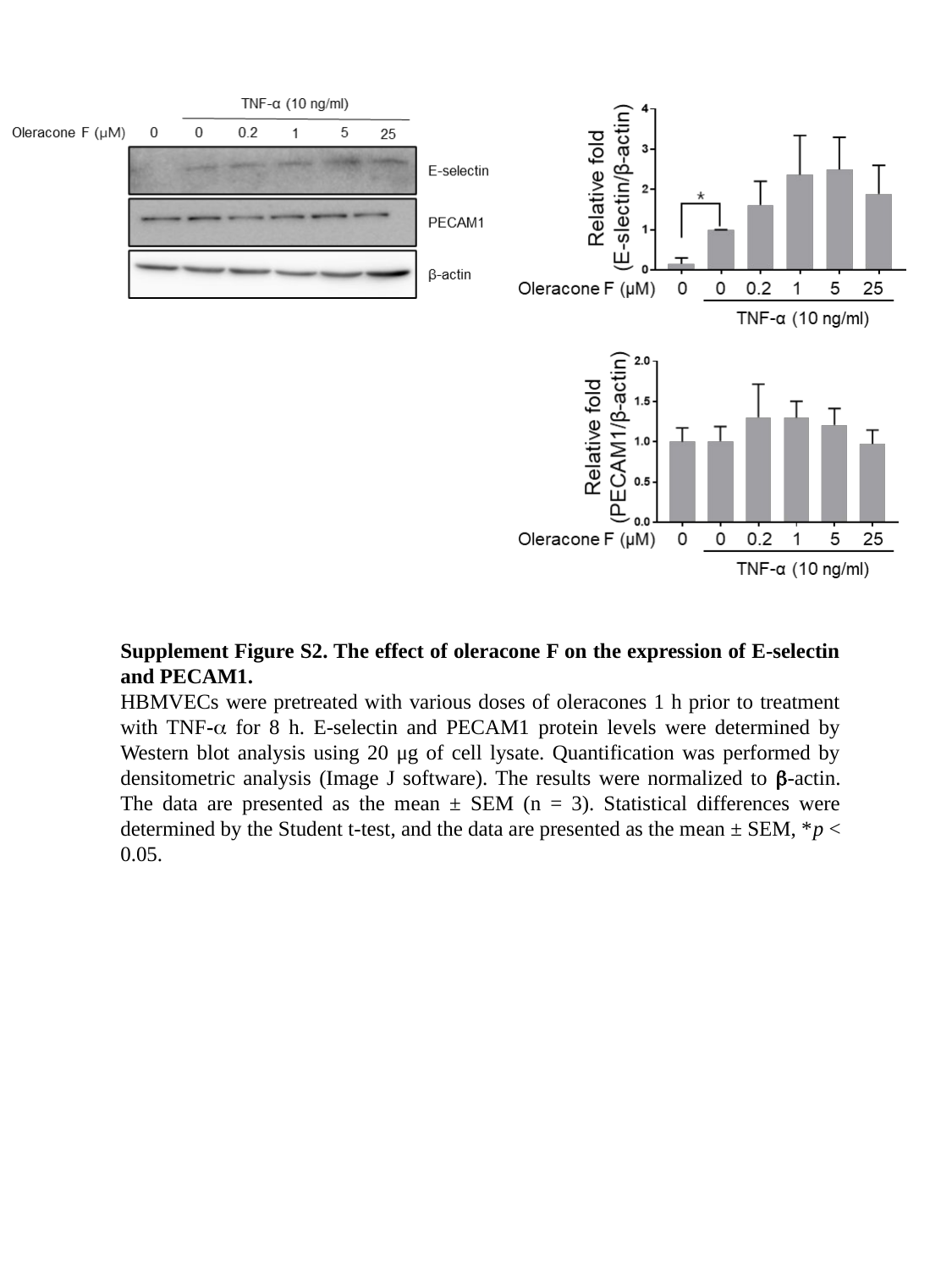

Supplement Figure S2. The effect of oleracone F on the expression of E-selectin and PECAM1.
HBMVECs were pretreated with various doses of oleracones 1 h prior to treatment with TNF-a for 8 h. E-selectin and PECAM1 protein levels were determined by Western blot analysis using 20 μg of cell lysate. Quantification was performed by densitometric analysis (Image J software). The results were normalized to b-actin. The data are presented as the mean ± SEM (n = 3). Statistical differences were determined by the Student t-test, and the data are presented as the mean ± SEM, *p < 0.05.
Supplement 1. The effect of oleracones on cell viability.
HBMVECs were pretreated with oleracones at the concentrations indicated 1 h prior to treatment with TNF-a (10 ng/mL, for 48 h). Cell viability was determined by the MTT assay.
